# First Report of Anterior Pallial Tentacles in *Solen dactylus* (Bivalvia: Solenidae) from the Northern Persian Gulf, Iran

**DOI:** 10.1371/journal.pone.0063487

**Published:** 2013-05-14

**Authors:** Hanieh Saeedi, Mark J. Costello, Rudo von Cosel

**Affiliations:** 1 Institute of Marine Science, University of Auckland, Auckland, New Zealand; 2 Muséum National d’Histoire Naturelle, Paris, France; Australian Museum, Australia

## Abstract

Solenidae are deep burrowing bivalves inhabiting intertidal and shallow sub-tidal soft bottom sediments mostly in tropical and sub-tropical areas. *Solen dactylus* has a restricted distribution within the Indian Ocean. *Solen dactylus* is frequently found on the sandy-muddy coast of the northern Persian Gulf, Iran. Specimens of *S. dactylus* were collected since 2006 from Bandar Abbas to study their biology and ecology. During these studies, an unexpected pair of anterior pallial tentacles at the dorsal end of the anterior pallial crest of the mantle was found. In the tentacles, two kinds of epithelial cells (pyramidal and vacuolated) and fibres (radial and longitudinal), and a branch of the pallial nerve located in the centre of a haemocoel, were determined. A possible coherence of a furrow parallel to the anterior shell margin with the presence of anterior pallial tentacles is discussed. All species with long anterior pallial tentacles have anterior shell furrows. Anterior pallial tentacles were found in 10 species of Solenidae from Asia to the Middle East and Europe. The function of the tentacles is unknown. However, more species need to be examined for anterior pallial tentacles and anterior shell furrows to determine if they reflect a common evolutionary history or ecology.

## Introduction

Razor shells and jackknife clams (Solenoidea: Solenidae and Pharidae) are a marine in-faunal bivalve superfamily. They inhabit soft bottoms in shallow water from the intertidal zone down to a depth of 110 m [1]. Razor clams have a long and strong foot which in Solenidae is more or less round and club-shaped and in Pharidae is laterally compressed and obliquely truncated. The foot enables the razor shells to burrow rapidly and deeply into the sediment using hydraulic power [2]. Solenidae are distributed in tropical and temperate seas worldwide [3], [4] with 68 accepted species [5].

As cephalic structures including sense organs have been lost in Bivalvia, siphonal and pallial tentacles have evolved. The mantle is fused except for the posterior siphonal openings and the anterior opening for the food. At the anterior pallial crest, a pair of anterior pallial tentacles may emerge. However, this special kind of anterior pallial tentacle has to date been found only in Solenidae and may be restricted to this family. They were first discovered by Morton (1988) in a small Indo-Pacific *Solen, S.* aff. *exiguus* Dunker, 1862 from Hong Kong. From histological transverse sections of the base and tip of the tentacles, Morton reported that basal squamous cells maintained the rigidity of the expanded tentacle, whereas in the tip only pyramidal and vacuolated cells were observed. He proposed that the anterior pallial tentacles are a specialized development of sensory papillae of the middle mantle fold surrounding the pedal gape [6]. Observations of such tentacles in several other *Solen* species from the Indo-West Pacific and Europe were published subsequently by Cosel (2002), Simone (2009) and Veeravaitaya (2010). It could be expected that anterior pallial tentacles are present in more *Solen* species. However, there have not been any other observations.

**Figure 1:**
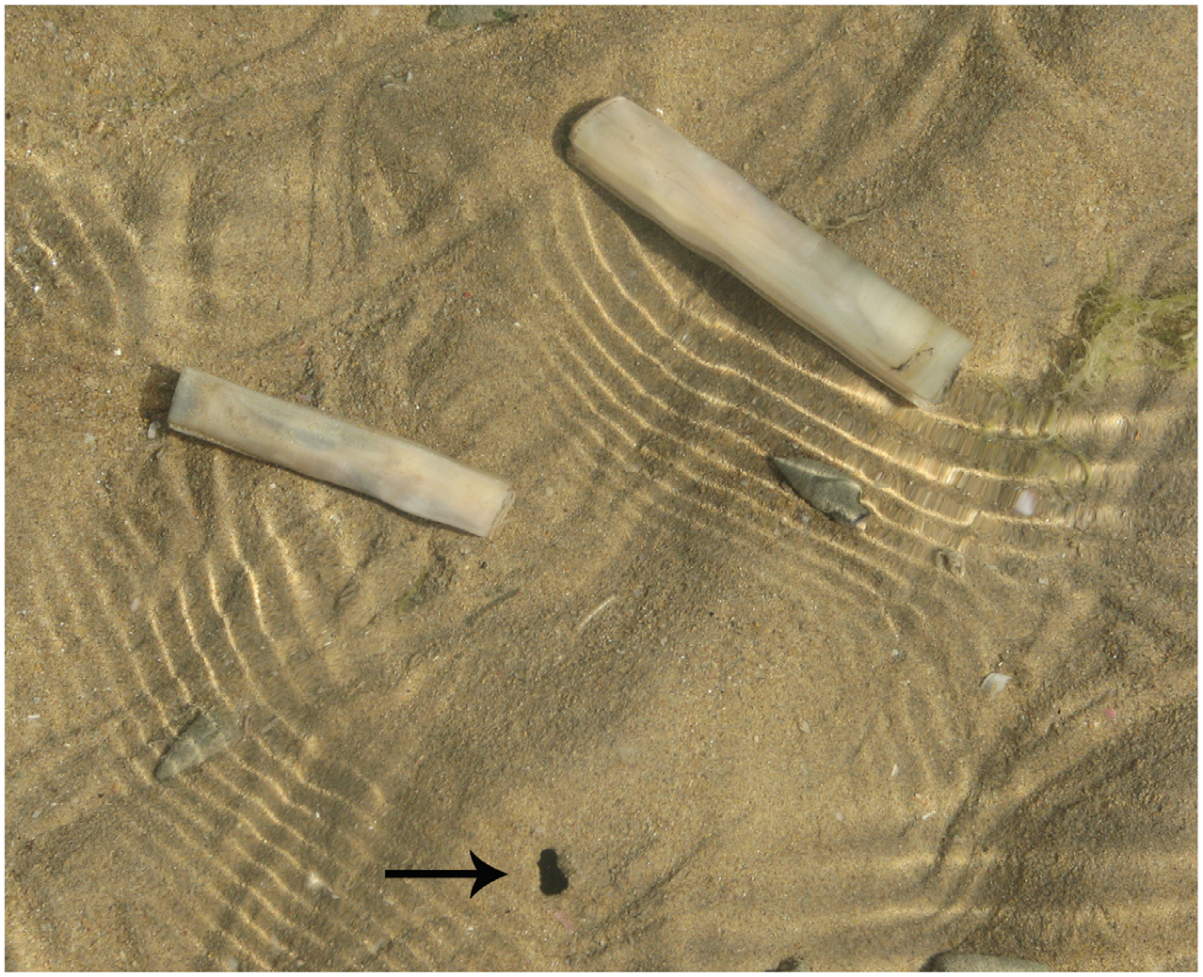
*Solen dactylus* Cosel, 1989 and its burrow (black arrow), Bandar Abbas, Iran.

**Figure 2:**
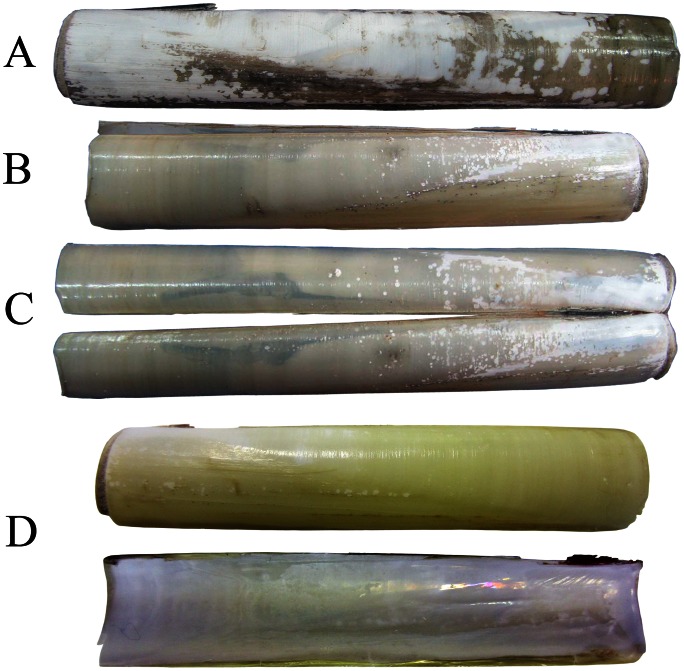
*Solen dactylus*, external [A, B, C, and upper D] and internal views [lower D] of the shell. All from Bandar Abbas, Iran; leg. H Saeedi.

**Figure 3:**
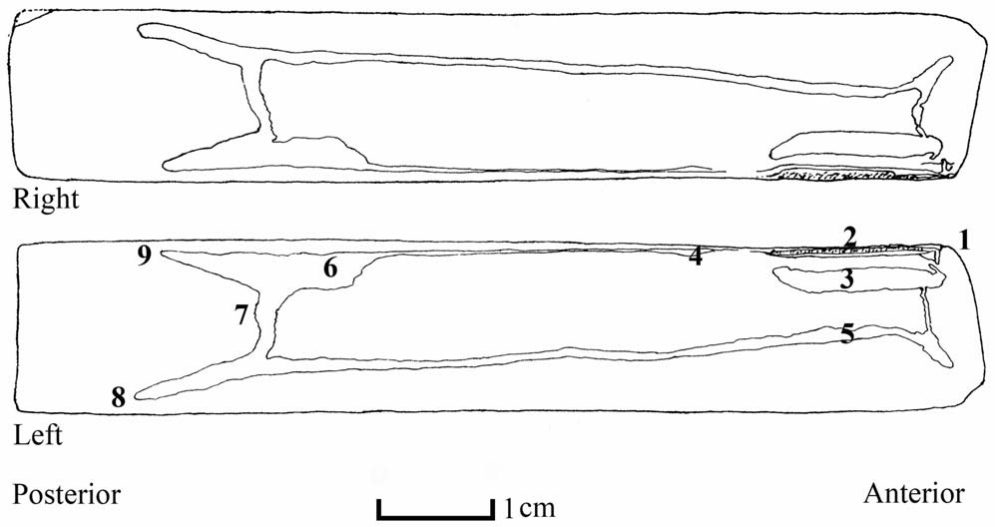
Internal view of both valves of *Solen dactylus*, half-schematic drawing. **1**: hinge; **2**: ligament; **3**: anterior adductor muscle scar; **4**: anterior retractor muscle scar; **5**: pallial muscle scar; **6**: posterior adductor muscle scar; **7**: pallial sinus; **8**: ventral limb of pallial sinus; **9**: dorsal limb of pallial sinus.

*Solen dactylus* Cosel, 1989 inhabits intertidal pools on sandy–muddy beaches, and occurs subtidally. It is endemic to the western Indian Ocean from the Gulf of Kutch (Kathiawar State, India) westwards along the Pakistan coast, the Oman Sea and into the inner Persian Gulf [2], [3], [7] (Figure 1). *Solen dactylus* has a white elongated and straight shell with parallel dorsal and ventral margins, one cardinal tooth in each valve and an external ligament (Figures 2 and 3) [2], [3], [8]. Herein, we report the first observation of anterior pallial tentacles in *S. dactylus*, consider their function, and review their occurrence in Solenidae.

## Materials and Methods

Specimens of *S. dactylus* were sampled from the southern coast of Bandar Abbas (e.g., Park-e Dolat, Park-e Qadir, and Nakhl-e Nakhoda), Iran from 2006 to 2012 [2], [9], [10]. In total, 1850 specimens were sampled (25–120 mm in total length and 0.3–23.0 g in total weight). No specific permits were required for the described field studies (locations) and specimens (razor clams). All locations were not privately-owned or protected in any way and did not involve endangered or protected species. Live specimens were observed under a binocular microscope immediately after sampling to record the movements of the anterior pallial tentacles. Then, samples were fixed either in 10% ethanol or formalin. Voucher specimens NO IM-2013-7127, IM-2013-7128, and IM-2013-7129 are in the Muséum National d’Histoire Naturelle of Paris. A small section of the tentacle near the tip was fixed in Bouin’s fixative for 24 h, preserved in 70% alcohol, dehydrated in an ethanol series and infiltrated with paraffin. Sections of 5 µm were cut and stained with haematoxylin and contrasted with eosin to identify the histological characters [2], [6].

## Results

**Figure 4:**
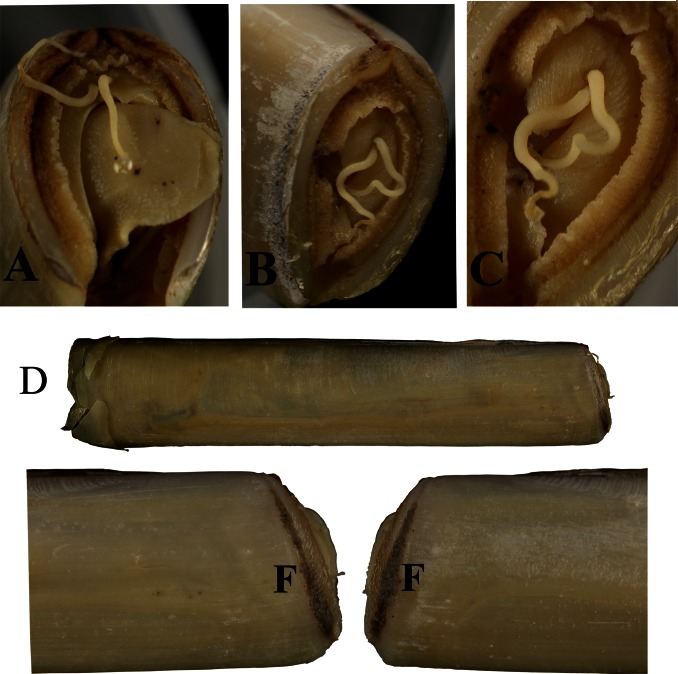
Anterior views of tentacle in two different specimens of *Solen dactylus* [A to C] and external view of the shell with animal [D]. Close-up view of anterior part of *Solen dactylus*, **F:** Furrow.

**Figure 5:**
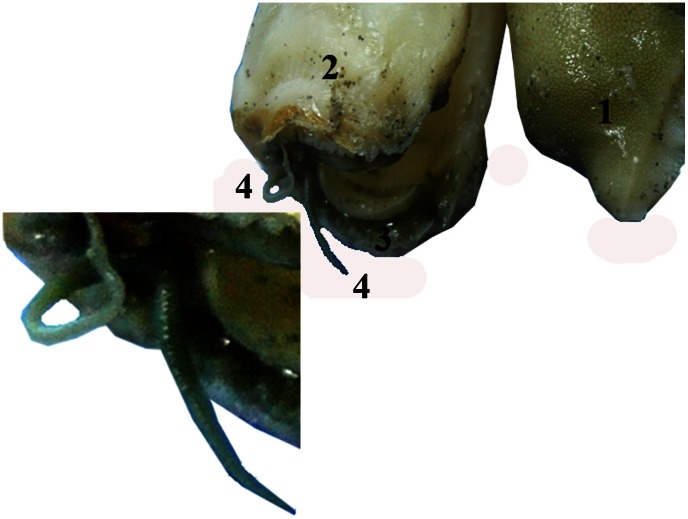
Anterior tentacles of a large specimen of *Solen dactylus* (with shell removed) attached to the mantle, 1: foot; 2: mantle; 3: pallial crest; 4: anterior tentacles.

The pair of pallial tentacles was located at the dorsal end of the anterior pallial crest, near the median line between the inner and middle mantle folds (Figures 4 and 5). The tentacles could be retracted or extended beyond the foot, curled, and bent.

**Figure 6:**
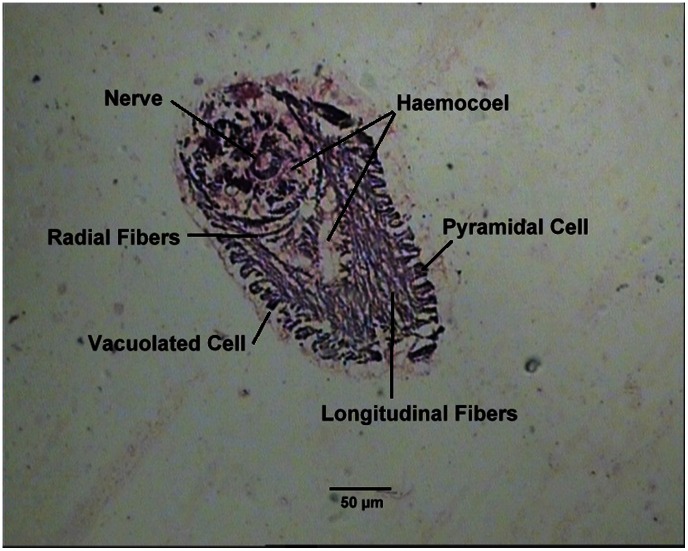
Transverse sections through a single anterior tentacle of *Solen dactylus*.

A transverse section of a tentacle showed two kinds of epithelial cells, pyramidal and vacuolated cells, and a central haemocoel. There was also an extensive network of radial and longitudinal fibres. A branch of the pallial nerve occurred in the centre of the haemocoel (Figure 6).

Table 1 lists all known occurrences of pallial tentacles in *Solen* species from East Asia, Iran or Europe. It is not known whether species of Solenidae from other regions have pallial tentacles or not. The tentacles have been found in subtidal and intertidal, and large and small, species (20–130 mm). At least 6 of the 10 species had a furrow along the anterior shell margin. The 3 species with long anterior pallial tentacles were found to have furrows, whereas the two species with short pallial tentacles were without furrows.

**Table 1:** *Solen* species reported to have anterior pallial tentacles.

Species	Depth zone	Substratum	Maximumdepth (m)	Shell length (mm)	Anteriortentacles	Anterior furrow	Reference
*Solen* cf. *canaliculatus*	Sub-tidal	Muddy sand	50	20–26	Small but rather long	Yes	Cosel, 2002
*Solen corneus*	Low tide to shallow water	Sandy muddy to fine sandy	No data	50–70	Very small	No	Cosel, 2002; Veeravaitaya, 2010
*Solen dactylus*	Intertidal	Sandy mud	10	65–120	Long and strong	Yes	Present Study
*Solen* aff. *exiguus*	Intertidal	Muddy sand	5	20	Short and small	No data	Morton, 1988
*Solen* cf. *exiguus*	Intertidal	Sand	10	32–35	Short and very small	No	Simone, 2009
*Solen kikuchii*	Intertidal to sub-tidal	Silty mud	50	24–26	Long and strong	Yes (slight furrow)	Cosel, 2002
*Solen marginatus*	Low tide to shallow water	Sandy mud	No data	65–130	Thin and rather short	Yes	Cosel, 2002
*Solen sarawakensis*	Shallow water	Sand	No data	87–134	Very small	Yes (filled furrow)	Cosel, 2002
*Solen thailandicus*	Low tide to shallow water	Sandy muddy to fine sandy	No data	35–49	Long and strong	Yes	Cosel, 2002
*Solen strictus*	Intertidal	No data	No data	100	Short and small	No data	Veeravaitaya, 2010

China and Thailand had the most *Solen* species with pallial tentacles (Table 2). No species were found in more than two countries. *Solen corneus* Lamarck, 1818 and *Solen strictus* Gould, 1861 have been observed in two different countries in Asia. However, only the Thailand populations of these two species have been reported to have anterior pallial tentacles. The only *Solen* species in Europe, *Solen marginatus*, has both tentacles and a furrow.

**Table 2:** Reported *Solen* species in regions where at least one species has pallial tentacles.

Species	China	Japan	Malaysia	Thailand	Taiwan	Iran	Europe
*Solen canaliculatus* Tchang & Hwang, 1964*	**+**						
*Solen* cf. *canaliculatus* Tchang & Hwang, 1964*					**+**		
*Solen corneus* Lamarck, 1818*			**+**	**+**			
*Solen dactylus* Cosel, 1989*						**+**	
*Solen* aff. *exiguus* Dunker, 1861*	**+**						
*Solen* cf. *exiguus* Dunker, 1861*				**+**			
*Solen kikuchii* Cosel, 2002*		**+**					
*Solen marginatus* Pulteney, 1799*							**+**
*Solen sarawakensis* Cosel, 2002*			**+**	**+**			
*Solen strictus* Gould, 1861*	**+**	**+**					
*Solen thailandicus* Cosel, 2002*				**+**			
*Solen gordonis*Yokoyama, 1920		**+**					
*Solen grandis* Dunker, 1862	**+**	**+**					
*Solen krusensterni* Schrenck, 1867		**+**					
*Solen kurodai* Habe, 1964		**+**					
*Solen linearis* Spengler, 1794	**+**		**+**				
*Solen pseudolinearis* Cosel, 2002	**+**						
*Solen regularis* Dunker, 1862			**+**	**+**			
*Solen roseomaculatus* Pilsbry, 1901	**+**	**+**					
*Solen sloanii* Gray, 1843	**+**	**+**					
*Solen soleneae* Cosel, 2002	**+**		**+**				
*Solen vagina* Linnaeus, 1758			**+**				

* Species reported to have pallial tentacles.

## Discussion

Studies of the presence or absence of the anterior palial tentacles still need to be improved. Only a few studies have considered the presence of the anterior pallial tentacles in *Solen* species. Cosel (2002) did not find anterior pallial tentacles in *Solen strictus* from Japan, *S. vagina* Linnaeus, 1758 from Malaysia or *Solen* sp. aff. *vagina* from Thailand. Although, Veeravaitaya (2010) reported anterior pallial tentacles in *S. strictus* from Don Hoi Lot, Thailand [11], this identification may be erroneous because *S. strictus* is a northern temperate species distributed along the coasts of Japan, Korea and far eastern Russia, but not tropical Southeast Asia such as Thailand.

As already mentioned, the tentacles are located at the dorsal end of the anterior pallial crest [6], [4], [12], [13] [present study]. The observed curling of the tentacles was “rather slow” for both *Solen canaliculatus* Tchang & Hwang, 1964 (Cosel, 2002) and *S. dactylus* (this study). Apparently the shell size does not affect the tentacles’ sizes. For example, *Solen kikuchii* Cosel, 2002 is small (25 mm) with very long tentacles, whereas the similarly sized species *S.* cf. *exiguus* Dunker, 1861 (32 mm) possesses short and small tentacles [12] (Table 1). Due to the absence of sufficient studies on the presence and function of the anterior pallial tentacles in *Solen*, it is not possible to consider them in a phylogeographic or ecological context (e.g., substratum or depth) (Table 1). However, the data (Table 1) suggest that the presence or absence of pallial tentacles is not related to the Solenidae body size, habitat or depth distribution.

It is possible that those Solenidae species having a furrow parallel to the anterior shell margin (Figure 4) also have a pair of anterior pallial tentacles. The furrows on the anterior end of the valves have different shapes, from deep to shallow and also from sharp and narrow (e.g., *Solen dactylus*) to very broad (e.g., *Solen canaliculatus*). Species of *Solen* with vertical furrows, of which the soft parts were examined, were found to have pallial tentacles: *Solen marginatus* Pulteney, 1799, *S. dactylus*, *S. thailandicus*, *S. canaliculatus*. However, other species with anterior pallial tentacles such as *S. corneus* and *S.* cf. *exiguus* lack an anterior furrow. *Solen kikuchii* has a slight constriction rather than a real furrow parallel to the anterior margin. *Solen sarawakensis* Cosel, 2002 has some kind of a “filled furrow”. When compared to the other species with a furrow, earlier layers of the anterior margin of the valves are well visible and not covered by later layers during growth. A filled furrow is almost flat and shows no real depression. Other species with anterior furrow are the Northern Australian *Solen darwinensis* Cosel, 2002 and *S. aureomaculatus* Habe, 1964, *S. vaginoides* Lamarck, 1818 from southern Australia, and *S. capensis* P. Fischer, 1881 from South Africa [14] (Table 1). It is possible that also in these species anterior pallial tentacles may be present. However, soft parts or live specimens of these have not yet been examined. Examination of as many Solenidae species as possible is required to elucidate the phylogeographic patterns in the occurrence of pallial tentacles.

Our results show that pallial tentacles are present in several species of Solenidae from Europe, the Middle East and the western Pacific. They may be present in other species in the Indo-west Pacific realm and East Africa but have not yet been observed. The functional role of the tentacles is also unclear. Morton (1988) suggested they may detect predators. However, why the foot could not do this, and what predators would be encountered deep in the burrow, is unknown. Experiments on the behaviour of living specimens may reveal this function. Study of the morphology, comparative anatomy and genetics of Solenidae species may also clarify the evolutionary origins of the pallial tentacles and the presence or absence of an anterior furrow, and whether the anterior tentacles are primitive or advanced features.
